# Chimeric Antigen Receptor (CAR) T Cell Therapy for Malignant Pleural Mesothelioma (MPM)

**DOI:** 10.3390/cancers9090115

**Published:** 2017-09-01

**Authors:** Astero Klampatsa, Andrew R. Haas, Edmund K. Moon, Steven M. Albelda

**Affiliations:** Division of Pulmonary, Allergy, and Critical Care, Department of Medicine, Perelman School of Medicine, University of Pennsylvania, Philadelphia, PA 19104, USA; Andrew.Haas2@uphs.upenn.edu (A.R.H.); Edmund.Moon@uphs.upenn.edu (E.K.M.); albelda@mail.med.upenn.edu (S.M.A.)

**Keywords:** immunotherapy, chimeric antigen receptor T cells, mesothelioma, adoptive cell transfer

## Abstract

Cancer immunotherapy has now become a recognized approach to treating cancers. In addition to checkpoint blockade, adoptive T cell transfer (ACT) using chimeric antigen receptors (CARs) has shown impressive clinical outcomes in leukemias and is now being explored in solid tumors. CARs are engineered receptors, stably or transiently transduced into T cells, that aim to enhance T cell effector function by recognizing and binding to a specific tumor-associated antigen. In this review, we provide a summary of CAR T cell preclinical studies and clinical trials for malignant pleural mesothelioma (MPM), a rare, locally invasive pleural cancer with poor prognosis. We list other attractive potential targets for CAR T cell therapy for MPM, and discuss augmentation strategies of CAR T cell therapy with other forms of immunotherapy in this disease.

## 1. Introduction

Malignant pleural mesothelioma (MPM) is a rare thoracic malignancy originating in the mesothelial cells of the pleural cavity. It is primarily caused by occupational or environmental inhalation exposure to asbestos, an abundant natural mineral with heat-resistance properties and low cost, which has been mined worldwide for over a century [1]. Following studies that linked asbestos with developing mesothelioma [2], stringent regulations regarding production and use of asbestos have been implemented in most Western countries. Nonetheless, the incidence of MPM continues to increase in some countries, reflecting the long latency period of 20–40 years [3]. While MPM incidence rates are currently increasing in Australia and the United Kingdom, they are declining in Japan [4,5]. The annual incidence of pleural mesothelioma in the United States is estimated to be around 3300 cases per year [6]. The US incidence rate peaked in 2000 and is now slowly declining, secondary to control of asbestos exposure. MPM is characterized by its locally aggressive phenotype and resistance to therapy. Current treatment options, including surgery, radiation, and chemotherapy, are inefficient in extending median survival to more than 9–17 months [7,8].

The immune system has been thought to play an important role in cancer surveillance and tumor rejection in humans [9], as evidenced by cases of spontaneous tumor regression, metastases regression after removal of the primary tumor, tumor infiltration by immune effector cells, and higher cancer incidence in immunocompromised patients [10]. This hypothesis has now been clearly corroborated in many tumors, including lung cancer, by the striking responses to immune checkpoint blockade [11].

The clinical impact of the immune microenvironment has also been implicated in MPM. In preclinical studies, regulatory T cells (Tregs) and immunosuppressive soluble factors promote MPM tumor growth by blunting any antitumor immune responses [12]. In an in vivo model of MPM, Hegmans et al. demonstrated that survival increases when FoxP3+CD4+CD25+ Tregs were depleted [12]. Adenosine and prostaglandin E2 (PGE2) immunosuppression can inhibit T cell function [13], and (cyclooxygenase-2) COX-2 inhibition does block the growth of small mesothelioma tumors through an immunological mechanism that appears to allow more effective cytotoxic T cell (CTL) accumulation in the tumors [14]. Less is known in human studies, but in MPM patients who have undergone surgical resection, the presence of a higher density of CD8+ tumor-infiltrating lymphocytes (TILs) in their tumors correlated significantly with better survival [10].

These findings stress the importance of understanding the dynamic associations between the pro-tumorigenic and anti-tumorigenic components of the MPM immune microenvironment. The interaction of these factors influences tumor growth, tumor progression, patient prognosis, and treatment response. This has led to the development of novel immunotherapeutic strategies aimed at activating the host’s immune system or overcoming components of the immunosuppressive tumor microenvironment. A number of recent clinical trials of immunotherapy, including intrapleural administration of an adenovirus expressing interferon alpha (Ad.IFNα), vaccination with a Wilm’s tumor-1 (WT-1) peptide analogue, the use of a dendritic cell vaccine, and anti-PD1 antibody treatment have suggested efficacy [15,16,17,18]. Our review describes the development and use of chimeric antigen receptor (CAR) T cells as an additional promising type of immunotherapy in the treatment of MPM, and describes the preclinical studies and clinical trials of CAR T cell therapy in MPM.

## 2. Adoptive Cell Therapy (ACT)

Adoptive Cell Therapy (ACT) is defined as the isolation of a patient’s own leukocytes (usually lymphocytes) that are expanded ex vivo, and then re-infused into to the patient. The concept behind ACT is that the re-infused tumor-reactive T cells will recognize and bind to tumor-associated antigens (TAAs) on the tumor cell surface, thus destroying the cancer cells. Early successes in melanoma patients were achieved by isolating autologous T cells from tumor biopsy specimens and rapidly expanding the most active TIL cultures screened for the greatest antitumor activity [19]. Rosenberg et al. found that ACT with autologous TILs mediated complete response in 22% patients with metastatic melanoma regardless any prior treatment [20]. Despite these successes, the use of TILs poses several challenges, including difficulty in their isolation and ex vivo expansion, and minimal clinical responses in other tumor types [21,22,23]. These obstacles have led to an increasing interest in genetically modifying peripheral blood T cells rather than expanding TILs.

## 3. Chimeric Antigen Receptors (CARs)

Chimeric antigen receptors are fused receptors engineered to provide antigen specificity to T cells against TAAs on the cell surface of target cells. CARs can bind directly to an expanded range of cell surface targets including lipids, carbohydrates, or proteins [24].

**Figure 1:**
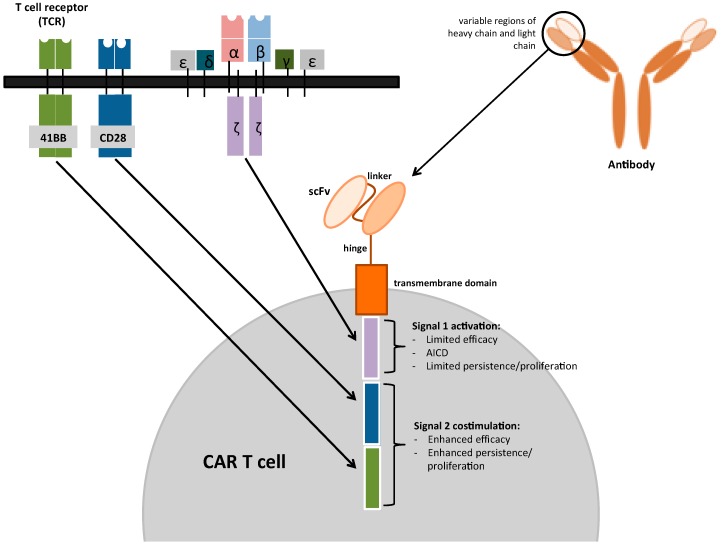
Chimeric antigen receptor (CAR) T cell. The single chain scFv—derived from the antigen-binding domain of antibodies—is fused to the CD3ζ transmembrane and intracellular signaling domains from the T-cell receptor complex; this is the first-generation CAR. Additional intracellular signaling domains are added for costimulatory signals, such as the CD28 and 4-1BB signaling domains, to yield second- and third-generation CARs.

“First-generation” CARs consisted of an extracellular domain that bound the tumor antigen via a single-chain variable antibody fragment (scFv) that was fused to a CD3ζ intracellular activating domain [25]. After antigen recognition on tumor cells, these CARs exhibited cytotoxic ability in vitro but had very limited activity in vivo due to the inability of the CD3ζ chain to adequately activate resting T cells [21,25]. To overcome this limitation, “second-generation” CARs were developed by fusing the scFv with a co-stimulatory intracellular signaling domain in tandem with the CD3ζ chain. Commonly incorporated co-stimulatory domains include CD28 and 4-1BB. These additional signaling domains led to enhanced cytokine production, increased T-cell proliferation and persistence, delayed apoptosis, and markedly improved anti-tumor efficacy in vivo. More recently, “third-generation” CARs have been developed that incorporate a CD3ζ domain, and two co-stimulatory domains within their cytoplasmic tail [25]. In preclinical studies, third-generation CARs have demonstrated superior antitumor efficacy compared with second-generation CARs [26,27]. The basic structure of CARs is shown in Figure 1.

Once constructed, CARs can be transduced into autologous T cells using viral (lentiviral or onco-retroviral) or non-viral (transposon) gene transfer systems [28] to achieve permanent CAR expression or using messenger RNA (mRNA) electroporation [29] to achieve transient expression for toxicity assessment. Following transduction, these CAR T cells can be expanded ex vivo in specialized GMP (Good Manufacturing Practice) facilities and re-infused to the patient, either systemically or regionally, as a therapeutic intervention. CARs targeting the B-cell antigen CD19, have shown dramatic results in clinical trials for a number of hematologic malignancies ((acute lymphoblastic leukemia (ALL), non-Hodgkin lymphoma (NHL), and chronic lymphocytic leukemia (CLL)) [30,31,32,33], and have provided the “proof of principle” rationale for CAR T cell development in a variety of solid tumors, including MPM.

A requirement for successful CAR T cell therapy, however, is a specific and highly expressed candidate TAA. In MPM, two such candidate target TAAs are currently being investigated in clinical trials: mesothelin, which is overexpressed on the tumor cells, and fibroblast activation protein (FAP) that is overexpressed on tumor stromal cells.

## 4. Mesothelin CARs

Overexpressed differentiation antigens are attractive targets for CAR T cell therapy because they are expressed at low levels on normal tissues while being pathologically expressed at high levels on cancer cells. Mesothelin is one such cell-surface glycoprotein that is expressed at low levels on normal mesothelial cells of the pleura, pericardium, peritoneum, and tunica vaginalis, whereas it is overexpressed in the majority of MPM, lung, pancreatic, and ovarian carcinomas [34]. Mesothelin is an especially appealing target since several preclinical and clinical studies have found that it is involved in the malignant transformation of tumors and has a clear association with tumor aggressiveness, which leads to local invasion and eventual metastasis [35,36].

Mesothelin CARs have been and are currently being investigated in multiple Phase I clinical trials (NCT02414269, NCT01583686, NCT02580747, NCT02159716, and NCT01355965). Based on potent anti-tumor effects observed in preclinical studies using mRNA electroporation [37], an initial study focusing on toxicity assessment was conducted (NCT01355965) at the University of Pennsylvania using T cells that only transiently expressed the second-generation anti-mesothelin CAR that contained the CD3ζ and 41BB signaling domains (7 mesothelioma patients and 7 pancreatic cancer patients) [29,38]. No patient in this Phase I safety trial demonstrated “on target, off tumor” toxicity (pleuritis, peritonitis, pericarditis) from CAR mesothelin T cell infusion; however, no consistent clinical responses were attained, however two patients showed some evidence of tumor shrinkage [29]. Interestingly, an immediate serious anaphylactic reaction was noted in one patient during the third mesothelin CAR T cell infusion that was attributed to the immunogenicity of the murine SS1 scFv used in the CAR construct [38]. Given the safety confirmation with transient CAR mesothelin expression, a second trial (NCT02159716) was conducted in patients with mesothelioma, ovarian cancer, and pancreatic cancer using a lentiviral transduction vector expressing the same murine-based anti-mesothelin second-generation CAR. In this trial (5 mesothelioma, 5 ovarian, and 5 pancreatic cancer patients), two different doses of T cells were administered, and cyclophosphamide was added as a lymphodepletion agent in some cohorts. The mesothelin CAR T cells were well tolerated and CAR T cells could be detected in the blood (using qPCR) for up to 30 days. Unfortunately, no clinical responses were reported (manuscript in preparation). A third trial has just been initiated using a more active, fully human anti-mesothelin CAR that should enhance persistence and efficacy (NCT03054298).

Investigators at the Memorial Sloan Kettering are also conducting an MPM clinical trial using mesothelin CAR T cells (NCT02414269). Their approach is based on preclinical studies in an orthotopic MPM mouse model showing potent and long-lasting antitumor efficacy of intrapleurally administered mesothelin CAR T-cell therapy [39]. This Phase I clinical trial uses a CAR with a human-derived anti-mesothelin scFv and a CD3z/CD28 signaling domain transduced using a retroviral vector and is being administered intrapleurally (rather than intravenously) in patients with primary or secondary pleural malignancies, with MPM being the primary target population. This intrapleural delivery approach may overcome the major hurdle of CAR T cell trafficking into tumor cells by directly administering the mesothelin CAR T cells to the tumors in the pleural space.

## 5. FAP CARs

**Figure 2:**
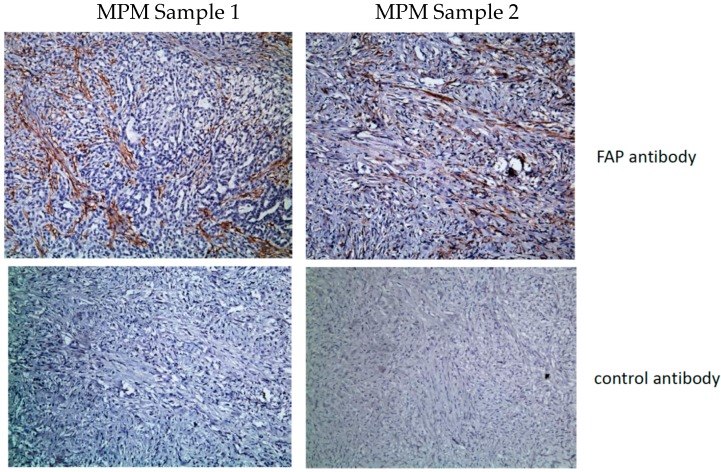
Fibroblast Activation Protein Staining in two malignant pleural mesotheliomas (MPMs). Two MPM paraffin-embedded samples were stained with an anti-FAP antibody (Abcam antibody #207178 (monoclonal rabbit IgG), 1:250 dilution) or an isotype control. Strong stromal staining (brown) is seen in both samples. (All micrographs at 10× magnification).

In addition to tumor-cell-specific antigens, it has been proposed that CARs that target essential components of the tumor-associated stroma, such as fibroblasts or endothelial cells, might also be valuable to enhance anti-tumor activity. There are multiple potential advantages to this approach: (1) stromal cells are more genetically stable and less likely to lose antigen expression, (2) attacking the stromal components may alter the tumor microenvironment to improve standard chemotherapeutic or ACT efficacy, and (3) it could be used on multiple different tumor types. Two proposed stromal candidates are fibroblast activation protein (FAP) and vascular endothelial growth factor receptor 2 (VEGFR2). FAP, a transmembrane serine protease, is highly expressed in the cancer-associated stromal cells (CASCs) of virtually all epithelial cancers with low expression on normal cells [40]. FAP is overexpressed in all three major MPM subtypes including epithelioid, sarcomatoid, and biphasic [41]. Figure 2 shows an example of FAP staining in two mesothelioma tumors.

Preclinical studies have demonstrated that CAR T cells targeted to murine FAP have anti-tumor efficacy in subcutaneous MPM models with minimal toxicity [40]. An anti-human directed FAP CAR with the CD3ζ and CD28 signaling domains was produced at the University of Zurich and shown to induce the killing of tumor cells expressing human FAP [41]. Based on these preclinical studies, this group has initiated a Phase I clinical trial to evaluate the safety of administering FAP-redirected T cells intrapleurally to patients with MPM (NCT01722149) [42].

## 6. Other CARs Targeting Potential Targets for MPM

### 6.1. Pan-ErbB “T4” CAR

In a recently published study, Klampatsa et al. investigated the efficiency of a CAR targeting the four members of the ErbB family (EGFR, HER2, ErbB3, and ErbB4) in MPM [43]. They firstly demonstrated expression of EGFR and ErbB4 in a cohort of MPM histological samples, which provided the rationale for testing this CAR in MPM [43]. To redirect T-cell specificity against the ErbB family, they engineered a second-generation CAR named T1E28z [43]. The CAR is co-expressed with a chimeric cytokine receptor named 4ab that delivers an interleukin (IL)-2/IL-15 signal upon binding of IL-4, thereby enabling the selective enrichment of CAR T cells during ex vivo expansion [43]. The study used MPM patients’ blood and showed that successful transduction and enrichment of CAR T cells was achieved in all patients, either at diagnosis or following chemotherapy. Functionality of the expanded cells was indicated both in vitro and in vivo, using an orthotopic peritoneal mesothelioma model [43]. These data provided support for the clinical evaluation of intra-cavitary T4 immunotherapy in MPM patients, and the group is currently seeking funding to commence a Phase I clinical trial.

### 6.2. 5T4 CAR

The oncofetal cell surface glycoprotein, 5T4, is overexpressed in numerous malignancies, including testicular, breast and colon cancer, while its expression in normal tissues is restricted to specialized epithelial cells [44]. Recently, it was reported that 5T4 was also expressed in a very high percentage of MPM cell lines and biopsies and pleural fluid samples. Interestingly, 5T4-specific CD8+ T cells were able to kill four out of six HLA-A2+ MPM cell lines but not an HLA-A2− cell line, demonstrating immune recognition of MPM-associated 5T4 antigen at the effector T-cell level [45]. Given that a 5T4 CAR has recently been generated and shown to efficiently kill 5T4-expressing nasopharyngeal carcinoma cells in vitro [46], 5T4 CARs represent an interesting potential future MPM therapeutic.

### 6.3. Chondroitin Sulfate Proteoglycan CARs

The cell surface proteoglycan chondroitin sulfate proteoglycan 4 (CSPG4) has been found to be overexpressed MPM where it has been reported that CSPG4 was overexpressed in six out of eight MPM cell lines, and in 25 out of 41 MPM biopsies [47]. In 2014, investigators from the National Cancer Institute in the United States constructed four second-generation CARs, each from a different murine monoclonal antibody, linked to the CD28 co-stimulatory domain and the intracellular T cell receptor signaling chain CD3ζ [48]. Donor T cells transduced with these CARs demonstrated cytokine release and cytolytic function when co-cultured with several tumor cell lines, including MPM [48]. The authors concluded that CSPG4 is an attractive target for CAR T-cell therapy, although some issues were raised about some low-level expression of this protein in normal small bowel samples [48].

### 6.4. The Future: CAR Augmentation Strategies

To date, the success of CAR T cells seen in hematologic malignancies has not yet been reproduced in solid tumors and successful MPM treatment with CAR T cells will doubtlessly be challenging. This will be due to multiple mechanisms that include poor trafficking of cells into the tumors, CAR T cell suppression due to soluble mediators (such as adenosine, TGFβ, and prostaglandin E2), the upregulation of checkpoint inhibitors (such as PD1) on the CARs, and CAR suppression due to intrinsic inhibitory T cell programs [49]. In addition, TAA expression heterogeneity and immune escape (due to antigen deletion) could also be potential issues.

Accordingly, many groups are already anticipating these problems and are developing approaches to overcome these hurdles. Strategies include systemic drugs that may affect immune function in general, such as the use of checkpoint blockade using antibodies [50], inhibitors of immunosuppressive agents, like indoleamine 2–3 dioxygenase (IDO) [51], adenosine, PGE2, or immunosuppressive cell types (like CD4 T-regulatory cells). CARs can also be combined with other types of immunotherapy such as oncolytic viruses or whole-cell vaccines [43].

The ability to genetically manipulate CARs allows the possibility of generating “better” CARs by inserting or removing specific genes, thus adding an additional level of opportunity and excitement. As examples, TGFβ inhibition has been blocked by expressing a decoy receptor [52], PD1-induced inhibition has been subverted by inserting a PD1-CD28 “switch receptor” that converts negative signals to stimulatory signals [53], the effects of adenosine and PGE2 have been inhibited by inserting a small peptide that prevents activation of protein kinase A [13], activating cytokines (like IL-12) can be delivered [54], trafficking can be enhanced by insertion of chemokine receptors [53], and CAR T cell function has been enhanced by using cytoplasmic domains derived from natural killer cells [55]. Many other approaches are being actively pursued.

## 7. Conclusions

With an ever-evolving understanding of tumor biology and the recent exciting advances in tumor immunology, the use of the body’s own defense systems to fight disease is more current and relevant than ever. The exciting successes seen in hematologic malignancies have prompted development of CAR T cell therapy for solid tumors, such as MPM. Clinical trials of CARs for use in mesothelioma are underway. The results of these trials will be used to iteratively improve the next series of trials, eventually leading, it is hoped, to adoptive T cell transfer as an important part of the MPM therapeutic armamentarium.
